# A Retrospective Pilot Study to Evaluate the Radiographic Prognosis of Calcified Carotid Atherosclerosis Based on Cone Beam Computed Tomography

**DOI:** 10.7759/cureus.95511

**Published:** 2025-10-27

**Authors:** Abrar A Alamoudi, Wazeer Alghamdi, Niranzena Panneer Selvam, Kahani R Soni, Andres Pinto, Faisal A Quereshy, Uma Irfan, Ali Z Syed

**Affiliations:** 1 Department of Oral Diagnostic Sciences, Faculty of Dentistry, King Abdul-Aziz University, Jeddah, SAU; 2 Department of Population and Quantitative Health Sciences, Case Western Reserve University School of Medicine, Cleveland, USA; 3 Department of Biomedical Dental Sciences, Imam Abdulrahman Bin Faisal University, Dammam, SAU; 4 Department of Oral Health Promotion, Creighton University School of Dentistry, Omaha, USA; 5 Department of Oral and Maxillofacial Medicine and Diagnostic Sciences, Case Western Reserve University School of Dental Medicine, Cleveland, USA; 6 Department of Oral and Maxillofacial Surgery, Case Western Reserve University School of Dental Medicine, Cleveland, USA

**Keywords:** calcified carotid atherosclerosis, calcium scores, cone-beam computed tomography, cone-beam computed tomography (cbct), heart disease

## Abstract

Background: Carotid atherosclerosis has been used to measure the risk for heart disease and stroke. With the use of cone beam computed tomography (CBCT), dentists can diagnose them earlier if the area is calcified. This study aims to assess the radiographic prognosis of calcified carotid atherosclerosis over time.

Methods: A retrospective pilot study including 50 patients with multiple CBCT scans was conducted. Two parameters were used to assess the prognosis of the calcification:(1) involvement of a new anatomical location on the second scan; and (2) a novel use of calcium scoring system by measuring the Agatston score (AGS), volume score, and density score utilizing CBCT data.

Results: Out of 50 patients, there were 28 (56%) females and 22 (44%) males. The mean age was 71±7.42. A slight increase in the number, size, and anatomical area of calcification was noted. There was a significant difference between the AGS on the left side and the volume score, P=0.0403, on the right side. The mean value of all scores on the second scan was higher than on the first.

Conclusions: Our study evaluated the progression of calcified carotid atherosclerosis in asymptomatic patients undergoing multiple CBCT scans. The findings indicate that there was a slight increase in the number, size, and anatomical area of calcification over time. Additionally, the introduction of a calcium scoring system allowed for more comprehensive assessment using the AGS, volume score, and density score derived from CBCT scan data.

## Introduction

The COVID-19 pandemic changed the mortality causes over the past years. However, it is a noteworthy fact that deaths in the United States attributed to heart disease and stroke retained their leading position in 2020 and 2021. This underscores the persistent and significant impact of cardiovascular diseases on public health despite the challenges posed by the ongoing pandemic, according to the National Center for Health Statistics (NCHS). Even previously, both conditions had the same rank in 2018 and 2019. Moreover, age-adjusted death rates increased by 4.1% for heart disease (161.5 to 168.2) and 4.9% for stroke (37.0 to 38.8). In parallel, the long-term care cost of stroke survival is also increasing significantly. It is estimated that in 2030, the cost would be triple that in 2012 [1]. Atherosclerosis of the carotid artery may be a treatable cause of ischemic stroke, as it is commonly used as a marker for assessing the risk of such events [2,3]. Literature shows diverse methods used to quantify the amount of carotid atherosclerosis and vessel patency, such as intimal-medial thickness, atherosclerotic plaque load and composition, and calcium scoring. According to Denzel et al., calcium scores of the calcified atherosclerosis are calculated by using computed tomography (CT) and allow precise quantification of the calcium content of atherosclerotic plaque in the carotid [4]. Early diagnosis or identification of the high-risk patient could improve the overall patient quality of life, reducing the mortality rate.

Dentists could play a role in identifying high-risk people early and thereby reducing cardiovascular risk. A regular dental checkup is usually based on patient risk, but patients mostly visit the dentist annually or biannually. Since caries is one of the most common infections, dentists will be encountering these patients more often than primary care providers and possibly at an earlier age [5,6]. Furthermore, cone beam computed tomography (CBCT) machines are becoming available in dental offices, and the carotid region could be captured in these scans, depending on the scan field of view. CBCT has benefitted dental patients by providing a more cost-effective and less radiation-intensive alternative to traditional CT scans.

Therefore, investigating how calcified atherosclerosis progresses with time could help in reducing the associated risk by early diagnosis and referral to a physician. This study aims to assess the radiographic prognosis of calcified atherosclerosis of the carotid artery over time. Two parameters will be used to assess the prognosis in the study: involvement of a new anatomical location in the second scan, which is not present in the first scan, and calculation of the calcium scoring system utilizing CBCT scan data.

## Materials and methods

Study design and ethics statement

A retrospective study was conducted in Case Western Reserve University School of Dental Medicine after obtaining ethical approval (STUDY20211507).

Study population and sample size

A total of 160 consecutive CBCT scans of patients with reported atherosclerosis, taken between January 1, 2019, and February 28, 2022, were assessed. Only patients with radiographic evidence of calcified atherosclerosis and with two different scans acquired over time were included. All scans were obtained using a CBCT machine (I-CAT FLX, Imaging Sciences International, Hatfield, PA, USA) with the following scan parameters: tube voltage, 120 kVp; tube current, 5 mA; exposure time, 3708 ms; field of view, 16 mm × 16 mm × 10 mm; and voxel size, 0.3 mm. Scans were excluded if patients had two scans acquired using different protocols, or if the scans were of poor quality or showed evidence of surgical intervention in the area of interest. After applying these criteria, the final sample size was 50. Most of the scans were originally obtained for the purpose of dental implant planning, followed by evaluation of dentoalveolar pathology.

Study measures

**Figure 1:**
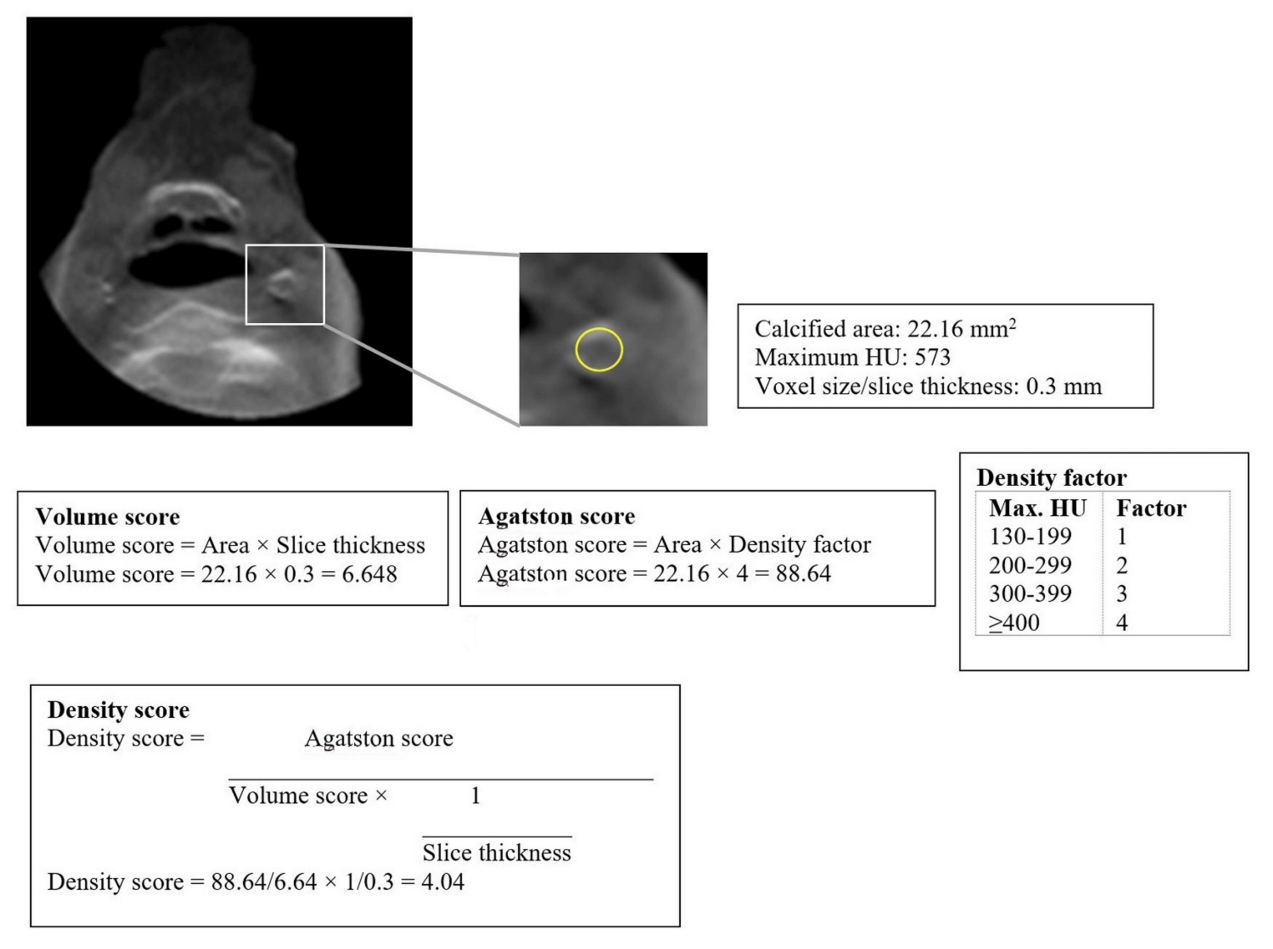
Calculation of volume, density, and AGSs AGS: Agatston score

Demographic data, social history, and radiographic interpretation were collected for each patient. Alcohol consumption data was collected based on the National Institute on Alcohol Abuse and Alcoholism. Based on the body mass index (BMI), patients were divided into four groups: 1, normal (18.5-24.9); 2, overweight (25-29.9); 3, obese (30-34.9), and 4, severely obese (>35). These grouping systems were based on medical consensus. Scans were evaluated by an oral and maxillofacial radiologist using the DICOM reader 3D Imaging Invivo ™ 6.0 (Anatomage, Inc., CA, USA). Since CBCT has poor soft tissue contrast, a novel method using a 3 mm axial slice thickness with an abdominal setting was used to mimic the soft tissue window of multidetector computed tomography (MDCT). Any carotid artery calcification areas were analyzed using the following criteria: (1) location of calcified atherosclerosis (right, left, or bilateral); (2) number of atherosclerotic areas on each side; (3) size of each area measured in millimeters (mm); (4) Agatston score (AGS), based on the calcification area and the density factor, where any area of calcium deposit with an attenuation >130 Hounsfield Units (HU) is multiplied by a density factor. The density factor is based on the maximum HU within the region of interest (grayscale values in CBCT were used to represent HU), with the maximum HU in the range of 130-199 assigned a factor of 1, 200-299 assigned a factor of 2, 300-399 assigned a factor of 3, and ≥400 assigned a factor of 4; (5) volume score, where the size of the calcification in CBCT is measured on the slice showing the largest calcification area, multiplied by the slice thickness; and (6) density score, calculated as the AGS divided by the volume score and multiplied by 1/slice thickness [7]. Figure 1 shows the calculation formulas.

Statistical analysis

MedCalc® Statistical Software version 20.211 (MedCalc Software Ltd, Ostend, Belgium; https://www.medcalc.org; 2023) was used to conduct all statistical analyses. Intra-examiner reliability for continuous variables was assessed using the Intraclass Correlation Coefficient (ICC) based on a two-way mixed-effects model with absolute agreement for single measures (ICC(A,1)). Intra-examiner reliability for categorical variables was assessed using Cohen’s Kappa (κ). Descriptive analyses using mean and standard deviation, or median and IQR, depending on the distribution. For group comparative analyses, repeated analysis of variance (ANOVA) test and the Kruskal-Wallis test were used as appropriate. When significant main effects were found, Bonferroni-corrected pairwise comparisons were performed to adjust for multiple testing. A p-value less than 0.05 was considered statistically significant.

## Results

Intra-examiner reliability

The ICC values were 0.996 (95% CI: 0.990-0.998) for right AGS, 0.990 (0.975-0.996) for right volume score, 0.992 (0.980-0.997) for left AGS, 0.993 (0.981-0.997) for left volume score, and 0.992 (0.981-0.997) for density score for both side, indicating excellent reliability across all parameters. Cohen’s Kappa coefficients demonstrated excellent intra-examiner reliability across categorical variables, with κ values ranging from 0.929 to 1.000 (p<0.001).

Descriptive study result

Out of 50 patients, 28 (56%) were female and 22 (44%) were male. The age range was from 43 to 82 years. Demographic data were missing for four samples. Our sample was primarily under the overweight category, with 22 samples (47.8%) under the low-risk drinking category. Six were active smokers, and 10 were prior smokers. The medical history showed that autoimmune disease, cardiac, and respiratory disease were the highest findings among our sample, with 17 patients (36.95%), 10 patients (21.74%), and nine patients (19.57%), respectively. A total of 65.22% of patients were taking cardiovascular medication, as shown in Table 1.

**Table 1:** General characteristics of patients in the study BMI: body mass index

Characteristic	Number (%)
Age (years) (mean±SD)	71±7.42
Gender	
Male	22 (44)
Female	28 (56)
BMI	
Normal	13 (28.9)
Overweight	16 (35.6)
Obese	11 (24.4)
Severely obese	5 (11.1)
Alcohol consumption	
Yes	22 (47.8)
No	24 (52.2)
Smoking history	
Non-smoker	30 (65.2)
Active smoker	6 (13.0)
Prior smoker	10 (21.7)

Due to the retrospective nature of this study, the time interval between the scans was not standardized and varied across our samples. The timing between scans depended on the clinical need of the scan and the availability of the patient, resulting in a heterogeneous distribution of inter-scan intervals. Exact intervals were recorded in Table 2 to support transparency and reproducibility.

**Table 2:** Time interval between scans

Time interval in months	N (%)
1-5	20 (40)
6-10	14 (28)
11-15	4 (8)
16-20	5 (10)
21-25	5 (10)
26-30	1 (2)
31-35	0 (0)
41	1 (2)

Analytical study result

The location of the calcification area increased in the common carotid artery bifurcation (CCA BI) by 6% over time, and the location of the calcification area increased in the internal carotid artery bifurcation (ICA BI) by 2%. Moreover, the single area of calcification increased to two regions by 6% over time.

Repeated-measures ANOVA demonstrated significant differences in AGS on the left side between the two scans, F=4.92, p=0.031. The assumption of sphericity was met (Greenhouse-Geisser ε=1.00; Huynh-Feldt ε=1.00). Mean scores increased from the first scan (M=43.4, SE=7.14, 95% CI (29.03, 57.75)) to the second scan (M=53.16, SE=6.88, 95% CI (39.34, 66.98)). Bonferroni-corrected pairwise comparisons confirmed that values from the second scan were significantly higher than those of the first scan, mean difference=9.77 (SE=4.40), p=0.031. A significant linear trend, t=2.22, p=0.031, further supported a consistent increase in AGS on the left side. Moreover, a significant main effect difference in performance of volume score was noted too, F=4.44, p=0.040. The assumption of sphericity was met (Greenhouse-Geisser ε=1.00; Huynh-Feldt ε=1.00). Therefore, the right volume scores increased from the first scan (M=344.29, SE=69.05, 95% CI (205.53, 483.05)) to the second scan (M=446.79, SE=97.00, 95% CI (251.85, 641.72)). Bonferroni-corrected pairwise comparisons confirmed that the second scan values were significantly higher than those of the first scan (mean difference=102.50, SE=48.67, p=0.040). A significant linear trend, t=2.11, p=0.040, further supported a consistent increase across scans. There was no other significant difference in the score between the scans. However, the mean on the second scan was higher in all the scores, as shown in Table 3.

**Table 3:** Comparison between scan 1 and scan 2 for AGS, density, and volume score ^†^P-values are derived from repeated-measures ANOVA test or Kruskal-Wallis test, depending on the distribution. ^*^P<0.05 was considered statistically significant. AGS: Agatston score; SE, standard error; ANOVA: analysis of variance

Variables	Scan 1 mean (SE)	Scan 2 mean (SE)	P-value^†^
AGS left	43.39 (7.14)	53.16 (6.88)	0.0312^*^
AGS right	44.73 (6.08)	47.65 (6.91)	0.4488
Density left	2.92 (0.24)	3.04 (0.23)	0.4789
Density right	2.99 (0.24)	3.04 (0.23)	0.7339
Volume left	376.07 (79.90)	427.03 (74.54)	0.1832
Volume right	344.29 (69.5)	446.79 (97.00)	0.0403^*^

There was no significant difference among all collected demographic data, except for the samples with different smoking habits. AGSs differed by smoking group (p=0.026) but did not change over time, with similar patterns across groups, indicating that smoking status influenced overall Agatston levels but not their change between scans. Volume scores differed by smoking group (p=0.001) and increased significantly over time (p=0.012), showing similar patterns across groups. This suggests that smoking status affected overall volume levels, while all groups exhibited comparable increases between scans. Sphericity assumptions were met, so no corrections were necessary. Additionally, density scores of the right carotid artery at scan 1 showed a trend toward higher values in smokers (2.5±1.9 vs 3.4±1.4, p=0.055). Sphericity assumptions were met for repeated measures, so no corrections were required, as shown in Table 4.

**Table 4:** Comparison between AGS, density, and volume in scan 1 and smoking history ^†^P-values are derived from repeated-measures ANOVA test or Kruskal-Wallis test, depending on the distribution. ^*^P<0.05 was considered statistically significant. AGS: Agatston score; SD: Standard deviation; ANOVA: analysis of variance

Smoking history	AGS right scan 1 (mean±SD)	Density right scan 1 (mean±SD)	Volume right scan 2 (mean±SD)
Non-smoker	35.8±39.3	2.5±1.9	311.0±471.5
Active smoker	94.0±54.9	4.0	1383.8±1310.5
Prior smoker	39.8±35.5	3.4±1.4	383.3±459.2
P-value^†^	(0.0422)^*^	(0.0547)^*^	(0.0519)^*^

Moreover, there was no significant difference among samples with different medical histories or cardiovascular medication, except between samples with various autoimmune diseases. AGSs differed significantly across autoimmune conditions (p=0.0431), with mean scores ranging from 8.8±10.9 to 127.5±9.3, as shown in Table 5. The density score showed a significant within-subjects effect (p<0.001), with changes across scans differing by autoimmune condition (p<0.001), indicating that the pattern of density changes over time varied between groups. Sphericity assumptions for the repeated-measures factor (density across the two scans) were met (Greenhouse-Geisser ε=1.000), so no corrections were required. These findings suggest that certain autoimmune conditions are associated with higher calcification levels. 

**Table 5:** Distribution of mean AGS in various autoimmune conditions ^†^P-values are derived from repeated-measures ANOVA test or Kruskal-Wallis test, depending on the distribution. ^*^P<0.05 was considered statistically significant. AGS: Agatston score; SD: Standard deviation; ANOVA: analysis of variance

Autoimmune disorder	AGS right (mean ± SD)
None	53.2±52.8
Unknown	8.8±10.9
Rheumatoid arthritis	127.5±9.3
Joint arthritis	25.6±25.7
HIV	46.6±37.4
Lupus	42.3
P-value^†^	(0.0431)^*^

## Discussion

The location of the calcification increased from unilateral presentation to bilateral by 6% in the extracranial carotid area and by 2% in the intracranial carotid area, meaning that the calcification location and area increased over time. This study was conducted to understand the prognosis of carotid artery calcification based on a CBCT scan. To our knowledge, this is the first study to compare the radiographic outcome alone. We utilized CBCT scans to conduct our study because we believe we screen and follow up with dental patients more often than medical patients. Often, multiple CBCT scans may be obtained based on the clinical judgment to provide appropriate care. This study was conducted to understand the prognosis of carotid artery calcification based on a CBCT scan.

Our study revealed that the right-sided carotid artery calcification was more than the left in patients with unilateral involvement. This finding was consistent with Selwaness M et al.'s study [8]. According to some studies, carotid plaque calcification contributes to the biomechanical stability of plaques. Atherosclerotic plaques with dense calcifications are less likely to rupture than those with less dense calcification, and therefore, they are less likely to cause symptoms [9]. However, the risk of ipsilateral ischemia and carotid artery calcification was correlated in a systematic review and meta-analysis published by Baradaran et al [10]. Moreover, some researchers have asserted that the presence of calcium within carotid plaques may serve as a separate diagnostic criterion for luminal stenosis and ischemic symptoms [11]. A hallmark of unstable plaque, according to recent research, is the calcified atherosclerotic load. Vessel wall calcification may occur at various stages and through different pathways during atherogenesis, which helps explain these inconsistencies. A recent report suggests that the stability of atheromatous plaques may be influenced by the type, quantity, and chemical composition of calcium. However, for us in dental practice, this is difficult to assess based on a CBCT scan alone [12].

A novel result in our study that was observed based on our results is that there is a significant difference only in the mean of the AGS on the left side and the volume score on the right side. However, the mean was higher among all the scores in the second scan than in the first. Despite the fact that atherosclerosis is considered a systemic disease, its distribution across the vascular system is not uniform, and it is believed to be influenced by a variety of factors, including vessel geometry. In addition, plaque severity and composition may vary depending on the location [8,13]. We saw similar results in our study. In the literature, it has been reported that plaque characteristics differ between the left and right carotid arteries. Left-sided hemorrhage, fibrous cap, and lipids were the most prevalent. Contrary to the left side, right-sided plaques primarily consist of calcification [8,14]. We think that with this difference in pattern in both carotid arteries, there may be differences in plaque thickness and composition due to geometric factors, such as the bifurcation angle or the configuration of the left carotid artery relative to the aortic arch, or because the left carotid artery directly connects to the aortic arch instead of the right carotid artery, which originates from the brachiocephalic artery. This anatomical variation will, in turn, affect the hemodynamic pressure. Previous studies already showed that the left carotid artery might be exposed to higher arterial pressures [8,15]. In addition to this, studies using B-mode ultrasound and MRI found that the left common carotid artery had a thicker intima-media than the right [8,16]. 

We found no significant difference in all findings between the two gender groups. Even though in the literature, men were more likely than women to have carotid atherosclerosis in most age groups until 75 years of age, ischemic stroke is associated with similar lifetime risks for men and women. Moreover, non-invasive imaging and histology have revealed differences in plaque morphology and composition between genders; women have greater stenosis than men in their carotid arteries, regardless of the age at which they develop stenosis [17]. However, plaque calcification was similar between both genders, as shown in our study [11,17,18]. Zhang et al. found that low BMI (less than 18) and those currently drinking were less likely to develop carotid atherosclerosis than the control group [19,20]. Contrary to Zhang et al., our findings showed no significant difference in BMI or alcohol habit. Additionally, our results are in line with the results of Zhang et al. and Gac. P et al. studies, which reported a significant difference between smokers and the amount of atherosclerosis [19,21].

There is no significant difference between medical history, cardiovascular medication, or atherosclerosis. However, in our study, we noted an association between calcium score and patients with autoimmune diseases. The autoimmune disease caused a significant difference in the AGS. This could be related to the variation in the amount of C-reactive protein in a different autoimmune condition, leading to variation in calcification. However, further study with a larger sample size may be required for further understanding of autoimmune disease and the C-reactive protein in relation to atherosclerosis. The adverse effects of cardiovascular disease and autoimmune conditions have been documented in the literature [22,23].

The limitation of this study is the use of grayscale (HU) values of the CBCT scan. A CBCT scan has poor soft tissue contrast. Despite this drawback, the grayscale (HU) was used in the literature for different clinical purposes. A strong correlation was determined between the grayscale of CBCT and HU of CT [24-30]. Several studies have attempted to establish a conversion formula between gray scale values and HU in CBCT and MDCT. However, these formulas are specific to the imaging parameters and the calibration phantoms used in the study and may not be generalizable to other imaging [29]. It is important to note that using grayscale values in CBCT for quantitative analysis should be done cautiously. These values can vary depending on the imaging parameters, such as the X-ray beam energy, the reconstruction algorithm, and the voxel size. In our study, we used the following criteria to address this issue: five random patients were selected to measure HU (each patient with two scans with the exact exposure protocols). The HU for the same areas was measured in both scans. The HU values were within a similar range. We followed these restricted inclusion criteria in our study. Aside from that, those scans were taken primarily for dental-related issues or maxillofacial pathology; therefore, the region of the carotid artery may not always be fully visualized in routine scans. The restricted inclusion criteria further restricted the study sample. Radiographic analysis was performed under optimal conditions, such as using high-resolution monitors and reduced ambient lighting conditions. These conditions may not be practical or consistently available in a typical dental practice setting. Lastly, this study did not control for potential confounding factors since the primary objective was to identify and report incidental carotid calcifications observed on CBCT scans rather than infer causality. These limitations highlight areas that could serve as hypotheses for future, more comprehensive studies.

## Conclusions

Our study evaluated the progression of calcified carotid atherosclerosis in asymptomatic patients who underwent multiple scans. The findings indicate a slight increase in the number, size, and anatomical distribution of calcifications over time. Additionally, the introduction of a novel application of the calcium scoring system may allow a more comprehensive assessment of carotid atherosclerosis using CBCT. Based on our data, we recommend referring patients for further evaluation of future cardiovascular risk, particularly those with a history of smoking or autoimmune disease.

## References

[1] Ovbiagele B, Goldstein LB, Higashida RT (2013). Forecasting the future of stroke in the United States: a policy statement from the American Heart Association and American Stroke Association. Stroke.

[2] (2022). Cerebrovascular disease: epidemiology and natural history. https://www.clinicalkey.es/.

[3] Murphy SL, Kochanek KD, Xu J, Arias E (2022). National Center for Health Statistics: Mortality in the United States, 2020. https://www.cdc.gov/nchs/products/databriefs/db427.htm.

[4] Denzel C, Lell M, Maak M (2004). Carotid artery calcium: accuracy of a calcium score by computed tomography-an in vitro study with comparison to sonography and histology. Eur J Vasc Endovasc Surg.

[5] Vieira AR, Babb LM (2012). Detection of carotid artery plaques in the dental setting. Open J Stomatol.

[6] Gustafsson N, Ahlqvist J, Näslund U (2020). Associations among periodontitis, calcified carotid artery atheromas, and risk of myocardial infarction. J Dent Res.

[7] Sandfort V, Bluemke DA (2017). CT calcium scoring. History, current status and outlook. Diagn Interv Imaging.

[8] Selwaness M, van den Bouwhuijsen Q, van Onkelen RS (2014). Atherosclerotic plaque in the left carotid artery is more vulnerable than in the right. Stroke.

[9] Kwee RM (2010). Systematic review on the association between calcification in carotid plaques and clinical ischemic symptoms. J Vasc Surg.

[10] Baradaran H, Al-Dasuqi K, Knight-Greenfield A (2017). Association between Carotid Plaque features on CTA and cerebrovascular ischemia: a systematic review and meta-analysis. AJNR Am J Neuroradiol.

[11] Wendorff C, Wendorff H, Pelisek J (2015). Carotid plaque morphology is significantly associated with sex, age, and history of neurological symptoms. Stroke.

[12] Yoon WJ, Crisostomo P, Halandras P, Bechara CF, Aulivola B (2019). The use of the Agatston calcium score in predicting carotid plaque vulnerability. Ann Vasc Surg.

[13] Odink AE, van der Lugt A, Hofman A, Hunink MG, Breteler MM, Krestin GP, Witteman JC (2007). Association between calcification in the coronary arteries, aortic arch and carotid arteries: the Rotterdam study. Atherosclerosis.

[14] Virmani R, Burke AP, Kolodgie FD, Farb A (2003). Pathology of the thin-cap fibroatheroma: a type of vulnerable plaque. J Interv Cardiol.

[15] Phan TG, Beare RJ, Jolley D (2012). Carotid artery anatomy and geometry as risk factors for carotid atherosclerotic disease. Stroke.

[16] Rodríguez Hernández SA, Kroon AA, van Boxtel MP, Mess WH, Lodder J, Jolles J, de Leeuw PW (2003). Is there a side predilection for cerebrovascular disease?. Hypertension.

[17] Gasbarrino K, Di Iorio D, Daskalopoulou SS (2022). Importance of sex and gender in ischaemic stroke and carotid atherosclerotic disease. Eur Heart J.

[18] Sangiorgi G, Roversi S, Biondi Zoccai G, Modena MG, Servadei F, Ippoliti A, Mauriello A (2013). Sex-related differences in carotid plaque features and inflammation. J Vasc Surg.

[19] Zhang C, Wang J, Ding S (2022). Relationship between lifestyle and metabolic factors and carotid atherosclerosis: a survey of 47,063 fatty and non-fatty liver patients in China. Front Cardiovasc Med.

[20] Mukamal KJ, Kronmal RA, Mittleman MA, O'Leary DH, Polak JF, Cushman M, Siscovick DS (2003). Alcohol consumption and carotid atherosclerosis in older adults: the Cardiovascular Health Study. Arterioscler Thromb Vasc Biol.

[21] Gać P, Jaźwiec P, Mazur G, Poręba R (2017). Exposure to cigarette smoke and the carotid arteries calcification index in patients with essential hypertension. Cardiovasc Toxicol.

[22] Wong ND, Budoff MJ, Ferdinand K (2022). Atherosclerotic cardiovascular disease risk assessment: an American Society for Preventive Cardiology clinical practice statement. Am J Prev Cardiol.

[23] Chung CP, Oeser A, Raggi P (2005). Increased coronary-artery atherosclerosis in rheumatoid arthritis: relationship to disease duration and cardiovascular risk factors. Arthritis Rheum.

[24] Giannelou M, Mavragani CP (2017). Cardiovascular disease in systemic lupus erythematosus: a comprehensive update. J Autoimmun.

[25] Parsa A, Ibrahim N, Hassan B, van der Stelt P, Wismeijer D (2015). Bone quality evaluation at dental implant site using multislice CT, micro-CT, and cone beam CT. Clin Oral Implants Res.

[26] Kaya S, Yavuz I, Uysal I, Akkuş Z (2012). Measuring bone density in healing periapical lesions by using cone beam computed tomography: a clinical investigation. J Endod.

[27] Patrick S, Birur NP, Gurushanth K, Raghavan AS, Gurudath S (2017). Comparison of gray values of cone-beam computed tomography with hounsfield units of multislice computed tomography: an in vitro study. Indian J Dent Res.

[28] Ludlow JB, Ivanovic M (2008). Comparative dosimetry of dental CBCT devices and 64-slice CT for oral and maxillofacial radiology. Oral Surg Oral Med Oral Pathol Oral Radiol Endod.

[29] Reeves TE, Mah P, McDavid WD (2012). Deriving Hounsfield units using grey levels in cone beam CT: a clinical application. Dentomaxillofac Radiol.

[30] Razi T, Emamverdizadeh P, Nilavar N, Razi S (2019). Comparison of the Hounsfield unit in CT scan with the gray level in cone-beam CT. J Dent Res Dent Clin Dent Prospects.

